# Genetic structure of red-handed howler monkey populations in the fragmented landscape of Eastern Brazilian Amazonia

**DOI:** 10.1590/s1415-47572010000400027

**Published:** 2010-12-01

**Authors:** Heitor B. Bastos, Evonnildo C. Gonçalves, Stephen F. Ferrari, Artur Silva, Maria Paula C. Schneider

**Affiliations:** 1 Laboratório de Polimorfismo do DNA, Departamento de Genética, Universidade Federal do Pará, Belém, PABrazil; 2 Departamento de Biologia, Universidade Federal de Sergipe, São Cristóvão, SEBrazil

**Keywords:** *Alouatta belzebul*, Amazonia, conservation, genetic structure, habitat fragmentation

## Abstract

We genotyped 15 microsatellite loci in order to evaluate the effects of habitat fragmentation, caused by flooding of the Tucuruí reservoir, on the genetic structure of *Alouatta belzebul* in eastern Amazonia. The analysis included two populations sampled in 1984, representing both margins of the Tocantins river, and three populations sampled 18 years later. Minimal differences in the diversity levels between present-day (Ho = 0.62-0.69 and A_R_ = 6.07-7.21) and pre-flooding (Ho = 0.60-0.62 and A _R_ = 6.27-6.77) populations indicated there was no significant loss of genetic variability, possibly because of successful management strategies applied during the flooding. The changes observed were limited to shifts in the composition of alleles, which presumably reflect the admixture of subpopulations during flooding. Given this, there were significant differences in the Rst values (p = 0.05) in all but one between-site comparison. Both present-day and original populations showed a deficit of heterozygotes, which suggests that this may be typical of the species, at least at a local level, perhaps because of specific ecological characteristics. The relatively large number of private alleles recorded in all populations may be a consequence of the Wahlund effect resulting from population admixture or a process of expansion rather than the loss of rare alleles through genetic drift. Additionally, the levels of genetic variability observed in this study were higher than those reported for other species of Neotropical primates, suggesting good fitness levels in these *A. belzebul* populations. Regular genetic monitoring of remnant populations, especially on islands, should nevertheless be an integral component of long-term management strategies.

## Introduction

Endemic to Brazil, the red-handed howler (*Alouatta belzebul*) is found in the southeastern Amazon basin and northern Atlantic Forest (Gregorin, 2006). Like other members of the genus (Di Fiore and Campbell, 2007), the red-handed howler is relatively tolerant of anthropogenic habitat disturbance (Bonvicino, 1989; Pinto *et al.*, 2003; Camargo CC, MSc Dissertation, Museu Paraense Emílo Goeldi, Belém, PA, 2005), although Atlantic Forest populations have been reduced to critical levels by deforestation. In contrast, Amazonian populations are relatively abundant, despite the comparatively high deforestation rates that affect the densely-populated southeastern basin (Lopes and Ferrari, 2000).

**Figure 1:**
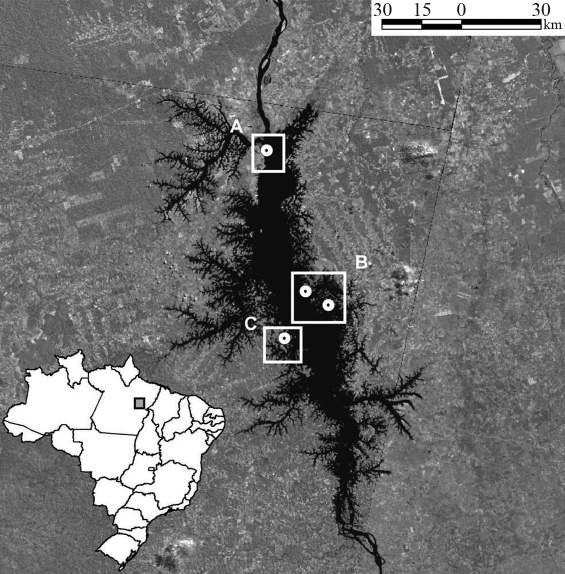
Map of the study area based on satellite imagery, showing the Tucuruí reservoir (center) and the sampling localities mentioned in the text. The Tocantins river (in black) runs south-north. In the surrounding matrix, darker gray areas are forest, while lighter coloration indicates pasture or plantations. A includes LB02 = Germoplasma Island in 2002, representing the left bank of the Tocantins post-flooding, B includes RB85 = Base 4 in 1985, representing the right bank prior to flooding, RB02 = Base 4 in 2002, representing the right bank post-flooding, and RB02B = Cornélio Island in 2002, an isolated post-flooding right bank population, C includes LB85 = Base 3 in 1985, representing the left bank prior to flooding of the reservoir.

Among other major impacts in this region, the construction in the early 1980s of the world's fourth-largest hydroelectric dam (based on generating capacity) at Tucuruí on the Tocantins river resulted in the creation of a 2500 km^2^ reservoir and the formation of more than 1600 islands. In addition to increasing the distance between opposite banks of the river from approximately 1 km to up to 30 km in places, construction of the dam initiated a process of intense human colonization in the area around the reservoir, which resulted in the fragmentation of most of the surrounding forest (Figure 1). Howlers were among the most abundant mammals prior to the flooding (Mascarenhas and Puorto, 1988), and currently have high population densities, especially on islands (Camargo and Ferrari, 2007), although the area's total population is almost certainly much smaller than it was prior to the flooding. Similarly, while there are no data on migration patterns, either before or after the flooding, it seems likely that dispersal is now restricted to the islands closest to each margin of the Tocantins and that cross-river transfer is practically nil.

Samples collected from some of the animals rescued during the flooding are still available, and the primary aims of the present study were to investigate the genetic structure of the original populations they represent, using molecular techniques not available in the 1980s, and to assess the long-term effects of the flooding on the structure of present-day populations. The isolation of relatively small populations on islands may lead to increased inbreeding and random effects such as genetic drift (Couvet, 2002; Frankham, 2003). These processes may cause a loss of genetic variability, and possible fixation of deleterious alleles, which in turn can lead to the loss of long-term population viability (Bjilsma *et al.*, 2000; Frankham *et al.*, 2002; Keller and Waller, 2002; Reed and Frankham, 2003).

## Material and Methods

###  Samples and DNA extraction

The samples analyzed in this study represent five spatially and temporally distinct populations of eastern Amazonian red-handed howlers (*Alouatta belzebul*) from the area of the Tucuruí hydroelectric reservoir (4°15'S, 49°31' W) in the Brazilian state of Pará (Figure 1). Blood samples (3-6 mL) were collected via venal puncture from 989 of the howlers captured during flooding of the reservoir, in 1984 and 1985. Aliquots of these samples were stored at the Universidade Federal do Pará in Belém. Most of these samples were used in the study of Schneider *et al.* (1991), but for the present study, it was only possible to extract DNA from 40 of the specimens. Of these, 30 were collected on the left bank (LB85) of the Tocantins and 10 on the right bank (RB85).

In 2002, howlers were captured live at three sites in the Tucuruí reservoir using a Pneu-dart rifle and tranquilizer darts containing 0.1 mL of ketamine hydrochloride per kg body weight. Captures were authorized by the federal environment agency (IBAMA) and were supervised by a veterinarian from the National Primate Center in Belém. All procedures were conducted in compliance with federal animal care legislation. Blood samples of ~ 3 mL were collected from the femoral vein of anesthetized howlers and stored in Falcon tubes containing 300 μL of 0.02 M EDTA. Thirty blood specimens were collected from howlers on the 129-ha Germoplasma Island (LB02), close to the present-day left bank of the Tocantins, and from 22 individuals on the right bank: 15 from Base 4 (RB02) and seven from Cornélio Island (RB02B; Figure 1), which is similar in size to Germoplasma Island. DNA was obtained from all samples using a standard protocol (Sambrook *et al.*, 1989).

###  Analysis of microsatellite loci

Fifteen microsatellite loci were analyzed (Table 1). Ten of these were developed specifically for *A. belzebul* (Gonçalves *et al.*, 2004), whereas the remaining five were specific for *Callithrix jacchus* (Nievergelt *et al.*, 1998), *Alouatta palliata* (Ellsworth and Hoelzer, 1998) and *Cebus apella* (Escobar-Páramo, 2000). Polymerase chain reactions (PCRs) were done in a final volume of 20 μL, containing 0.5 unit of *Taq* DNA polymerase, 5 ng of DNA, 25 μM of each dNTP, 50 mM KCl, 10 mM Tris-HCl and 0.4 μM of each primer. Optimal conditions for the 15 primers were determined by varying the temperature of association (Table 1), number of cycles and the concentration of MgCl_2_. The standard PCR profile was: an initial cycle of 5 min at 94 °C for denaturation, followed by 20-30 cycles of 1 min at 94 °C for denaturation, 1 min at 50-60 °C for primer association and 1 min at 72 °C for extension, with a final incubation of 5 min at 72 °C to guarantee complete extension of the PCR products. The PCR products were separated by size in 8% polyacrylamide gels in an ALF Express II automatic DNA sequencer (Amersham Bioscience). The size of the amplified fragments was determined using Allelinks 1.0 software.

###  Detection of bottleneck events

Recent bottleneck events often present genetic characteristics that can be detected in microsatellite data. Typically, allelic diversity is lost more quickly than heterozygosity, and observed heterozygosity is different from that expected based on the number of alleles under mutation-drift equilibrium (Cornuet and Luikart, 1996; Luikart and Cornuet, 1998). Allele frequencies were tested for the presence of an excess of heterozygotes using the sign test (Cornuet and Luikart, 1996) and Wilcoxon's signed-rank test, run on the Bottleneck program described by Piry *et al.* (1999). As the mechanisms of mutation in microsatellite markers are unknown, these tests were based on three different mutational models: the stepwise mutation model (SMM) (Kimura and Ohta, 1978), the infinite allele model (IAM) (Kimura and Crow, 1964), and the two-phase model (TPM) (Di Rienzo *et al.*, 1994). The TPM was used in conjunction with 95% SMM and 5% IAM, which, according to Piry *et al.* (1999), is appropriate for microsatellite markers.

###  Statistical analysis

To characterize the genetic diversity of each population, allele frequencies, the mean number of alleles (A), observed (Ho) and expected (He) heterozygosities according to Hardy-Weinberg equilibrium, and their means, were calculated using Popgene32 (Yeh *et al.*, 1997). Allelic richness (A_R_), an index of the number of alleles independent of sample size, was estimated using the Goudet (1995) FSTAT application via the rarefaction method adapted for genetic data by Petit *et al.* (1998). Possible deviations from Hardy-Weinberg equilibrium and an excess or deficiency of heterozygotes in a given population were evaluated using the exact probability test run on Genepop 3.1 (Raymond and Rousset, 1995). Genetic differentiation between populations was evaluated based on estimates of Rst, which is analogous to Fst (Wright, 1931). Arlequin 3.1 (Schneider *et al.*, 2000) was used to calculate Rst and run an analysis of molecular variance (AMOVA) (Excoffier *et al.*, 1992), which estimates the proportion of overall variance attributed to differences within and among artificial groups of the populations, and among individuals within each population. This analysis was done on all possible combinations of the five populations studied, although only the results (after Bonferroni correction; Rice, 1989) of the four most informative arrangements are presented here.

## Results

###  Genetic variability and bottlenecks

Some of the loci, such as Cj12 and Cj14, were relatively homogeneous in terms of allele length, whereas others, in particular Ab12 and Ab20, were more variable (Table 1). The mean number of alleles per locus was relatively large in all populations, ranging from 6.1 at RB02B to 12.0 at LB02; estimates of allelic richness varied less, from 6.07 at RB02B to 7.21 at LB02 (Table 2).

Observed heterozygosity was relatively high (≥ 0.60) in all populations (Table 2), although these values were consistently lower than expected (He ≥ 0.78) for each population. All differences between the observed and expected heterozygosities were significant, even after applying the Bonferroni correction (Rice, 1989). This indicates that none of the populations was in Hardy-Weinberg equilibrium.

The results of the Bottleneck analyses (Table 3) varied considerably, depending on the mutational model used and the type of statistical test. In the case of the IAM, the analyses indicated that some populations (LB85, LB02 and RB02B) had or have suffered recent bottleneck events. However, this model is more appropriate for protein studies, and when the SMM and TPM were applied, this trend disappeared. In the case of SMM, the sign test yielded a significant result for LB85, although this was contradicted emphatically by the Wilcoxon value (Table 3). Overall, these analyses indicated that none of the populations was experiencing or had experienced a recent bottleneck event.

###  Genetic differentiation

Values of pairwise Rst varied from -0.0109 (RB85 *vs.* RB02) to 0.2277 (LB85 *vs.* RB02B) (Table 4). With the exception of the comparison RB85 *vs.* RB02 (the same site in different years), all comparisons were significant (p < 0.05), and those involving RB02B generally returned the highest values, as expected from the relative configuration of the sites, *i.e.*, LB02 was more similar to LB85 than RB85, and so on. Despite being the most differentiated population, the Rst values for RB02B were consistent with its location in time and space, with smaller values for the RB populations and the smallest for the more recent RB02.

Other patterns were more complex, however, in particular the relatively low values recorded for RB02. Indeed, the negative value obtained for RB85 *vs.* RB02 suggested that there was more differentiation within each population than between them. RB02 was nevertheless more similar to both LB populations than it was to RB02B. This almost certainly reflects the admixture resulting from the release of rescued animals at Base 4, in contrast to Cornélio Island, which has remained isolated since the flooding.

###  Analysis of molecular variance (AMOVA)

Within-population variation accounted for > 90% of the molecular variance in all combinations of populations, except where RB02B was compared with the remaining populations (Table 5). In all other cases, the absence of variation between banks and the reduced variation among populations of the same bank was due to the inclusion of RB02B in the analysis.

## Discussion

###  Genetic variability

A relatively large number of alleles was recorded per locus compared with other platyrrhines, *e.g.Leontopithecus rosalia* (Grativol *et al.*, 2001) and *Mico argentatus* (Gonçalves *et al.*, 2009). Despite these values, there was considerable variation among populations, which may be accounted for primarily by differences in sample size. Indeed, Leberg (2002) has suggested that estimates of allele numbers in genetically variable populations are relatively more vulnerable to differences in sample size than those for more homogeneous populations. In addition, Schlötterer *et al.* (2004) have shown that mean allele excess may be strongly influenced by sample size and θ-values, and this seems to be the case with our data (not shown).

Unfortunately, the allelic richness parameter has not been used widely in studies of platyrrhines, which reduces its value for interspecific comparisons, although it does permit more conclusive inferences on differences among the populations studied here, without the bias of sample size. A comparison of allelic richness values (Table 2) indicated that overall the populations were relatively similar.

The heterozygosity values recorded here reconfirmed the relatively high genetic variability of the *A. belzebul* populations from Tucuruí, both before and after flooding of the reservoir. Once again, these values are also relatively high when compared with those for other microsatellite studies in platyrrhines: 0.49 (range: 0.34-0.65) in *L. rosalia* (Grativol *et al.*, 2001), 0.26 (0.11-0.39) in *M. argentatus* (Gonçalves *et al.*, 2009), 0.33 (0.09-0.65) in *C. moloch* (Menescal *et al.*, 2009) and 0.47 (0-0.86) in *Alouatta palliata* (Milton *et al.*, 2009).

The observed heterozygosity was significantly lower than expected in all five populations. However, this may be related to sampling questions, such as the Wahlund effect. In this case, the situation in the original populations (LB85 and RB85) might be explained by the admixture of different local subpopulations during flooding, although it seems likely that these populations, especially those on islands, have been relatively stable since then. However, the 18 years that separate the two periods represent only two or three howler generations (Di Fiore and Campbell, 2007), which may not yet have been sufficient for the populations to achieve equilibrium.

###  Differences among populations

The Rst values (Table 4) indicated relatively limited differentiation among populations, except for comparisons involving Cornélio Island. By comparison, Grativol *et al.* (2001) obtained values between 0.25 and 0.45 for pairwise comparisons of four isolated populations of *L. rosalia* in a fragmented habitat. In particular, the similarity between the left and right bank populations of *A. belzebul* suggests that the original course of the Tocantins river was not a very effective barrier to dispersal between the region's original populations. This might be expected, given the preference of Amazonian howlers for flooded forest ecosystems (Queiroz, 1995; Peres, 1997) and the original topography of the area, which was characterized by a complex of fluvial islands.

The negative value recorded for RB85 *vs.* RB02 is consistent with the fact that the latter represents the same location on the right bank of the Tocantins at a different point in time. The Rst value recorded for this comparison emphatically reinforces that this population has not suffered significant changes in its genetic structure. This may have been reinforced by the fact that, since howlers may survive to up to 20 years in the wild (Wang and Milton, 2003), at least part of the 2002 population may have been present at the site before the flooding. However, few data are available on the longevity of howlers in the wild (and none on *A. belzebul*), although studies at other sites have indicated a maximum life span of much less than 10 years (Di Fiore and Campbell, 2007), which would preclude this possibility.

The more pronounced differences between Cornélio Island and all other populations (confirmed by the Rst values and AMOVA results) may reflect the relative isolation of this island population. It is possible that formation of the island led to the isolation of a relatively differentiated local subset of the right bank population, which was not sampled in 1985, and has been reinforced by its subsequent isolation.

###  Implications for conservation of the species

Overall, the populations of *A. belzebul* show relatively ample genetic variability, with little evidence of significant bottleneck events or loss of variability in comparison with the original, pre-flooding populations. The changes observed were limited to shifts in allelic composition, presumably because of the admixture of local subpopulations during flooding. The phenotypic effects of inbreeding are not apparent at the moment, although the relatively small populations on islands are likely to be susceptible to inbreeding and genetic drift over the long term (Frankham, 1998).

The relatively large number of private alleles recorded in all populations (Table 2) may be a consequence of the Wahlund effect resulting from population admixture, a conclusion supported by the linkage disequilibrium observed in some pairs of loci (not shown). Alternatively, this excess of private alleles may reflect a process of expansion rather than the loss of rare alleles through genetic drift, particularly since rapid population growth enhances the retention of new mutations (Avise *et al.*, 1984; Rogers and Harpending, 1992). Since genetic variability is essential for the adaptation of a species to novel environments (Frankham, 2003), this parameter is an important indicator of the relatively good genetic viability of the populations, at least in the short term.

Various studies (*e.g.* Chiarello, 2003; Ferrari *et al.*, 2003; Gilbert, 2003; Rodriguez-Toledo *et al.*, 2003; Camargo CC, MSc Dissertation, Museu Paraense Emílo Goeldi, Belém, PA, 2005) have shown that howlers are not only highly tolerant of habitat disturbance, but may actually thrive in fragmented landscapes. Despite their ecological flexibility, which is based on folivory, howlers are not exempt from the long-term effects of the loss of genetic diversity or the accumulation of deleterious alleles. Tucuruí is unusual because of the effective isolation of fragments (= islands) by water, rather than a terrestrial matrix, across which howlers may disperse more easily. Given this, the regular monitoring of the genetic variability of these populations, especially those on islands, should be an integral component of long-term management strategies.

## Figures and Tables

**Table 1:** The 15 microsatellite loci analyzed in this study.

Locus	Temp (ºC)	Original species	Allele length (bp)
Ab04	62	*Alouatta belzebul*	147-167
Ab06	50	*Alouatta belzebul*	265-293
Ab07	60	*Alouatta belzebul*	178-202
Ab09	53	*Alouatta belzebul*	168-204
Ab10	56	*Alouatta belzebul*	228-284
Ab12	60	*Alouatta belzebul*	223-293
Ab13	52	*Alouatta belzebul*	197-261
Ab16	58	*Alouatta belzebul*	198-244
Ab17	63	*Alouatta belzebul*	193-263
Ab20	67	*Alouatta belzebul*	242-304
Cj12	50	*Callithrix jacchus*	120-134
Cj14	50	*Callithrix jacchus*	138-148
Ap68	49	*Alouatta palliata*	180-206
Ap74	52	*Alouatta palliata*	128-158
Pepc8	60	*Cebus apella*	224-246

**Table 2:** Summary of the characteristics of 15 microsatellite loci in the *A. belzebul* populations studied at Tucuruí, and analysis of the deviation from Hardy-Weinberg equilibrium.

Population (N)	A (SD)	A_R_	P_A_	Ho (SD)	He (SD)	p
LB85 (30)	11.27 (4.93)	6.77	20	0.60 (0.27)	0.83 (0.09)	< 0.01
RB85 (10)	6.93 (2.46)	6.27	3	0.67 (0.32)	0.79 (0.16)	< 0.01
LB02 (30)	12.00 (4.26)	7.21	37	0.69 (0.20)	0.86 (0.07)	< 0.01
RB02 (15)	8.40 (4.01)	6.26	9	0.62 (0.28)	0.78 (0.23)	< 0.01
RB02B (07)	6.07 (2.25)	6.07	5	0.67 (0.25)	0.78 (0.21)	< 0.05

See Figure 1 and text for definition of populations. A = mean number of alleles per locus, A_R_ = allelic richness, He = expected heterozygosity, Ho = observed heterozygosity, N = sample size, p = probability of deviation from Hardy-Weinberg equilibrium, P_A_ = private alleles, SD = standard deviation.

**Table 3:** Values of p recorded for the statistical analyses (sign and Wilcoxon tests) of the different mutational models used to evaluate the probability of recent bottleneck events in the study populations, calculated using the Bottleneck program of Piry *et al.* (1999).

	Value of p according to							
	Infinite alleles model			Stepwise mutation model			Two-phase model	
Population	Sign test	Wilcoxon test		Sign test	Wilcoxon test		Sign test	Wilcoxon test
LB85	0.00043	0.00002		0.01191	0.95837		0.42747	0.68066
RB85	0.38652	0.15140		0.10613	0.87381		0.09296	0.83487
LB02	0.00550	0.00005		0.23770	0.80530		0.42810	0.61923
RB02	0.00858	0.00009		0.44556	0.40387		0.27522	0.33490
RB02B	0.12625	0.07571		0.59758	0.33939		0.22683	0.24435

See Figure 1 and text for definition of populations.

**Table 4:** Matrix of Rst values for pairwise comparisons between *A. belzebul* populations from Tucuruí.

Population	LB85	RB85	LB02	RB02
RB85	0.0484*			
LB02	0.0592**	0.1001**		
RB02	0.0370*	-0.0109	0.0504**	
RB02B	0.2277**	0.1170*	0.1994**	0.1090*

See Figure 1 and text for definition of populations. *p < 0.05; **p < 0.01.

**Table 5:** AMOVA results for the principal arrangements of the *A. belzebul* populations from Tucuruí.

Arrangement	Between groups	Among populations within each group	Within populations
LB85 - RB85 *vs.* RB02B - LB02 - RB02	-1.13%	8.90%	92.23%
LB02 *vs.* LB85 - RB85 - RB02B - RB02	-0.68%	8.54%	92.14%
RB02 *vs.* LB85 - RB85 - LB02 - RB02B	-5.52%	10.43%	95.09%
RB02B *vs.* LB85 - RB85 - LB02 - RB02	14.76%	4.35%	80.89%

See Figure 1 and text for definition of populations.

## References

[Aviseetal1984] Avise J.C., Neigel J.E., Arnold J. (1984). Demographic influences on mitochondrial DNA lineage survivorship in animal populations. *J Mol Evol*.

[Bjilsmaetal2000] Bjilsma R., Bundgaard J., Boerema A.C. (2000). Does inbreeding affect the extinction risk of small populations? Predictions from *Drosophila*. J Evol Biol.

[Bonvicino1989] Bonvicino C.R. (1989). Ecologia e comportamento de *Alouatta belzebul* (Primates, Cebidae) na Mata Atlântica. Rev Nordest Biol.

[CamargoandFerrari2007] Camargo C.C., Ferrari S.F. (2007). Interactions between tayras (*Eira barbara*) and red-handed howlers (*Alouatta belzebul*) in eastern Amazonia. Primates.

[Chiarello2003] Chiarello A.G., Marsh L.K. (2003). Primates of the Brazilian Atlantic Forest: The influence of forest fragmentation on survival. Primates in Fragments.

[CornuetandLuikart1996] Cornuet J.M., Luikart G. (1996). Description and power analysis of two tests for detecting recent population bottlenecks from allele frequency data. Genetics.

[Couvet2002] Couvet D. (2002). Deleterious effects of restricted gene flow in fragmented populations. Conserv Biol.

[DiFioreandCampbell2007] Di Fiore A., Campbell C.J., Campbell C.J., Fuentes A., MacKinnon K.C., Panger M., Bearder S.K. (2007). The atelines: Variation in ecology, behavior, and social organization. Primates in Perspective.

[DiRienzoetal1994] Di Rienzo A., Peterson A.C., Garza J.C., Valdes A.M., Slatkin M., Freimer N.B. (1994). Mutational processes of simple-sequence repeat loci in human populations. Proc Natl Acad Sci USA.

[EllsworthandHoelzer1998] Ellsworth J.A., Hoelzer G.A. (1998). Characterization of microsatellite loci in a New World primate, the mantled howler monkey (*Alouatta palliata*). Mol Ecol.

[Escobar-Paramo2000] Escobar-Páramo P. (2000). Microsatellite primers for the wild brown capuchin monkey *Cebus apella*. Mol Ecol.

[Excoffieretal1992] Excoffier L., Smouse P.E., Quattro J.M. (1992). Analysis of molecular variance inferred from metric distances among DNA haplotypes: Application to human mitochondrial DNA restriction data. Genetics.

[Ferrarietal2003] Ferrari S.F., Iwanaga S., Ravetta A.L., Freitas F.C., Sousa B.A.R., Souza L.L., Costa C.G., Marsh L.K. (2003). Dynamics of primate communities along the Santarém-Cuiabá highway in south-central Brazilian Amazonia. Primates in Fragments.

[Frankham1998] Frankham R. (1998). Inbreeding and extinction: Island populations. Conserv Biol.

[Frankham2003] Frankham R. (2003). Genetics and conservation biology. C R Biol.

[Frankhametal2002] Frankham R., Ballou J.D., Briscoe D.A. (2002). Introduction to Conservation Genetics.

[Gilbert2003] Gilbert A.K., Marsh L.K. (2003). Primates and fragmentation of the Amazon forest. Primates in Fragments.

[Goncalvesetal2004] Gonçalves E.C., Silva A., Barbosa M.S.R., Schneider M.P.C. (2004). Isolation and characterization of microsatellite loci in Amazonian red-handed howlers *Alouatta belzebul* (Primates, Plathyrrini). Mol Ecol Notes.

[Goncalvesetal2009] Gonçalves E.C., Ferrari S.F., Coutinho P., Menezes E.V., Silva A., Schneider M.P.C., Davis L.C., Ford S.M., Porter L.M. (2009). Limited dispersal and genetic structure of silvery marmosets (*Mico argentatus*) in the fragmented landscape of central Amazonia. The Smallest Anthropoids: The Marmoset/Callimico Radiation.

[Goudet1995] Goudet J. (1995). FSTAT v. 1.2: A computer program to calculate F-statistics. J Hered.

[Grativoletal2001] Grativol A.D., Ballou J.D., Fleischer R.C. (2001). Microsatellite variation within and among recently fragmented populations of the golden lion tamarin (*Leontopithecus rosalia*). Conserv Genet.

[Gregorin2006] Gregorin R. (2006). Taxonomia e variação geográfica das espécies do gênero *Alouatta* Lacépède (Primates, Atelidae) no Brasil. Rev Bras Zool.

[KellerandWaller2002] Keller L.F., Waller D.M. (2002). Inbreeding effects in wild populations. Trends Ecol Evol.

[KimuraandCrow1964] Kimura M., Crow J.F. (1964). The number of alleles that can be maintained in a finite population. Genetics.

[KimuraandOhta1978] Kimura M., Ohta T. (1978). Stepwise mutation model and distribution of allelic frequencies in a finite population. Proc Natl Acad Sci USA.

[Leberg2002] Leberg P.L. (2002). Estimating allelic diversity: Effects of sample size and bottlenecks. Mol Ecol.

[LopesandFerrari2000] Lopes M.A., Ferrari S.F. (2000). Effects of human colonization on the abundance and diversity of mammals in eastern Brazilian Amazonia. Conserv Biol.

[LuikartandCornuet1998] Luikart G., Cornuet J.M. (1998). Empirical evaluation of a test for identifying recently bottlenecked populations from allele frequency data. Conserv Biol.

[MascarenhasandPuorto1988] Mascarenhas B.M., Puorto G. (1988). Nonvolant mammals rescued at the Tucuruí dam in the Brazilian Amazon. Primate Conserv.

[Menescaletal2009] Menescal L.A., Gonçalves E.C., Silva A., Ferrari S.F., Schneider M.P.C. (2009). Genetic diversity of red-bellied titis (*Callicebus moloch*) from eastern Amazonia based on microsatellite markers. Biochem Genet.

[Miltonetal2009] Milton K., Lozier J.D., Lacey E.A. (2009). Genetic structure of an isolated population of mantled howler monkeys (*Alouatta palliata*) on Barro Colorado Island, Panama. Conserv Genet.

[Nievergeltetal1998] Nievergelt C.M., Mundy N.I., Woodruff D.S. (1998). Microsatellite primers for genotyping common marmosets (*Callithrix jacchus*) and other callitrichids. Mol Ecol.

[Peres1997] Peres C.A. (1997). Effects of habitat quality and hunting pressure on arboreal folivore densities in Neotropical forests: Acase study of howler monkeys (*Alouatta* spp. ). Folia Primat.

[Petitetal1998] Petit R.J., El Mousadik A., Pons O. (1998). Identifying populations for conservation on the basis of genetic markers. Conserv Biol.

[Pintoetal2003] Pinto A.C.B., Ramos C.A., Carvalho O. (2003). Activity patterns and diet of the howler monkey *Alouatta belzebul* in areas of logged and unlogged forest in eastern Amazonia. Anim Biodiv Conserv.

[Piryetal1999] Piry S., Luikart G., Cornuet J.M. (1999). BOTTLENECK: A computer program for detecting reductions in the effective size using allele frequency. J Hered.

[Queiroz1995] Queiroz H.L. (1995). Preguiças e Guaribas: Os Mamíferos Folívoros Arborícolas do Mamirauá.

[RaymondandRousset1995] Raymond M., Rousset F. (1995). GENEPOP v. 1.2: Population genetics software for exact tests and ecumenicism. J Hered.

[ReedandFrankham2003] Reed D.H., Frankham R. (2003). Correlation between fitness and genetic diversity. Conserv Biol.

[Rice1989] Rice W.R. (1989). Analyzing tables of statistical tests. Evolution.

[Rodriguez-Toledoetal2003] Rodriguez-Toledo E.M., Mandujano S., Garcia-Orduña F., Marsh L.K. (2003). Relationships between forest fragments and howler monkeys (*Alouatta palliata mexicana*) in southern Vera Cruz, Mexico. Primates in Fragments.

[RogersandHarpending1992] Rogers A.R., Harpending H. (1992). Population growth makes waves in the distribution of pairwise genetic differences. Mol Biol Evol.

[Sambrooketal1989] Sambrook J., Fritsch E.F., Maniatis T. (1989). Molecular Cloning: A Laboratory Manual.

[Schlottereretal2004] Schlötterer C., Kauer M., Dieringer D. (2004). Allele excess at neutrally evolving microsatellites and the implications for tests of neutrality. Proc Biol Sci.

[Schneideretal1991] Schneider H., Sampaio M.I.C., Schneider M.P.C., Ayres J.M., Barroso C.M.L., Hamel A.R., Silva B.T.F., Salzano F.M. (1991). Coat color and biochemical variation in Amazonian wild populations of *Alouatta belzebul*. Am J Phys Anthropol.

[Schneideretal2000] Schneider S., Roessli E., Excoffier L. (2000). Arlequin v. 3.11: A software for population genetic data analysis.

[WangandMilton2003] Wang E., Milton K. (2003). Intragroup social relationships of male *Alouatta palliate* on Barro Colorado Island, Republic of Panama. Int J Primatol.

[Wright1931] Wright S. (1931). Evolution in mendelian population. Genetics.

[Yehetal1997] Yeh F.C., Yang R.C., Boylet T. (1997). POPGENE v. 1.32: Software Microsoft Windows based freeware for population genetic analysis.

